# Integrated Small RNA Sequencing, Transcriptome and GWAS Data Reveal microRNA Regulation in Response to Milk Protein Traits in Chinese Holstein Cattle

**DOI:** 10.3389/fgene.2021.726706

**Published:** 2021-10-12

**Authors:** Wentao Cai, Cong Li, Junya Li, Jiuzhou Song, Shengli Zhang

**Affiliations:** ^1^ Laboratory of Molecular Biology and Bovine Breeding, Institute of Animal Science, Chinese Academy of Agricultural Sciences, Beijing, China; ^2^ Key Laboratory of Animal Genetics, Breeding and Reproduction, Ministry of Agriculture & National Engineering Laboratory for Animal Breeding, College of Animal Science and Technology, China Agricultural University, Beijing, China; ^3^ Department of Animal and Avian Science, University of Maryland, College Park, MD, United States

**Keywords:** microRNA, transcriptome, GWAS, milk protein, holstein, milk production traits

## Abstract

Milk protein is one of the most important economic traits in the dairy industry. Yet, the regulatory network of miRNAs for the synthesis of milk protein in mammary is poorly understood. Samples from 12 Chinese Holstein cows with three high ( ≥ 3.5%) and three low ( ≤ 3.0%) phenotypic values for milk protein percentage in lactation and non-lactation were examined through deep small RNA sequencing. We characterized 388 known and 212 novel miRNAs in the mammary gland. Differentially expressed analysis detected 28 miRNAs in lactation and 52 miRNAs in the non-lactating period with a highly significant correlation with milk protein concentration. Target prediction and correlation analysis identified some key miRNAs and their targets potentially involved in the synthesis of milk protein. We analyzed for enrichments of GWAS signals in miRNAs and their correlated targets. Our results demonstrated that genomic regions harboring DE miRNA genes in lactation were significantly enriched with GWAS signals for milk protein percentage traits and that enrichments within DE miRNA targets were significantly higher than in random gene sets for the majority of milk production traits. This integrated study on the transcriptome and posttranscriptional regulatory profiles between significantly differential phenotypes of milk protein concentration provides new insights into the mechanism of milk protein synthesis, which should reveal the regulatory mechanisms of milk secretion.

## Introduction

Milk protein is one of the best protein sources for humans (Anderson et al., 2004). It also affects milk-manufacturing properties such as cheese yields, milk coagulation time and curd firmness (Auldist et al., 2004; Wedholm et al., 2006). Improving milk protein yields and quality can increase the economic outcome of the dairy industry. It has been reported that the amount and compositions of proteins in milk are determined mainly by genetic factors (Auldist et al., 2004). The heritabilities of milk protein compositions were moderate to high in Dutch Holstein-Friesian cattle, ranging from 0.25 to 0.80 (Schopen et al., 2009). So far, several strategies, such as QTL mapping, candidate gene analysis, genome-wide association studies (GWAS), or next-generation sequencing (NGS) technologies (Georges et al., 1995; Gambra et al., 2013; Zhou et al., 2019), have been adopted to identify several causal genes and mutations associated with increased protein yield and composition. However, the synthesis and secretion of milk proteins involve complex physiological and biochemical processes. One of these mechanisms is related to the role of microRNAs (miRNAs), which need to be thoroughly examined.

MiRNAs are a class of small (18–24 nucleotide) RNAs that are involved in the regulation of gene expression by targeting messenger RNAs (mRNAs). The vast majority of miRNA genes are transcribed by the RNA polymerase II, which generates long primary transcripts (pri-miRNA) that contain a hairpin stem-loop structure (Lee et al., 2003). miRNAs are processed from double-stranded hairpin precursors by Drosha protein in the nucleus and Dicer protein in the cytoplasm. The final single-stranded mature miRNA hybridizes with the RNA-induced silencing complex (RISC) to undergo gene inhibition (Robb and Rana, 2007; Kim et al., 2009). Unlike other regulators, miRNAs exert highly complex and combinatorial regulations by targeting hundreds of mRNA transcripts (Shivdasani, 2006). Extensive research in the past decade indicates the involvement of miRNAs in various biological processes such as cell development, proliferation, differentiation and apoptosis (Johnson et al., 2005; Gu and Iyer, 2006; Zhou et al., 2007). Recently, miRNAs have been shown to play important roles in the milk secretion process through their altered regulation of genes involved in milk protein and fat synthesis (Wang et al., 2016; Cui et al., 2020). Fifty-six mammary miRNAs were reported to have significant differences in expression between the lactation and non-lactating periods (begins 60 days before the expected calving) in Holstein cows (Li et al., 2012b). Several miRNAs, such as miR-15a (Li et al., 2012a), miR-139 (Cui et al., 2017), miR-423-5p (Mahmoudi et al., 2015), miR-101b (Tanaka et al., 2009), miR-486 (Li et al., 2015a), miR-152 (Wang et al., 2014), miR-135 (Ji et al., 2015) and miR-138 (Li et al., 2012b), appear to affect milk protein synthesis by regulating key genes of protein synthesis pathways. Although the identification and characterization of miRNA in bovine have been reported (Li et al., 2012b; Le Guillou et al., 2014; Li et al., 2015b; Wang et al., 2016), to our knowledge, only a few studies describe miRNA profiles specific to the synthesis of milk protein in bovine. The inspiration of many miRNA studies in milk protein synthesis in bovine was extrapolated, some even from another biological process that was unknown in mammary tissue before (Li et al., 2015a; Cui et al., 2017). The real miRNA profiles specific to milk protein traits are limited in bovine.

In this study, the hypothesis was that miRNAs have potential roles in milk protein production of cattle. Using miRNA-seq and RNA-seq, we investigated the miRNA profiles of mammary glands from 12 Chinese Holstein cows with three high (≥3.5%) and three low (≤3.0%) phenotypic values for milk protein percentage in lactation and non-lactating period. We believe that the results from the integrated transcriptome analyses of miRNA, mRNA and GWAS signals will help us identify new miRNA related to milk protein, further enhancing our understanding of the mechanisms of milk protein synthesis.

## Materials and Methods

### Ethics approval and consent to participate

All animal experiments were performed following the recommendations in the Guide for the Care and Use of Laboratory Animals of China. The study protocol was approved by the College of Animal Science 98 and Technology, China Agricultural University (Permit Number: DK996).

### Mammary Samples

The 12 multiparous (second to fourth parities) and healthy Chinese Holstein cows with three too high and three low phenotypic values for milk protein percentage peak and non-lactation period were chosen from the Beijing Sanyuan Dairy Farm Center (Beijing, China), which has been described in the previous study (Li et al., 2016). In brief, the cows were maintained in free stall housing and were fed a total mixed ration (TMR) with *ad libitum* access to water. We defined a high milk protein percentage group as those cows with 3.5% protein and the low milk protein percentage group was composed of cows with 3.0% protein throughout the previous lactation based on Dairy Herd Improvement system (DHI) data (Supplementary Table S1). Six cows were selected at approximately 79 days postpartum (peak lactation) and the other six cows during the non-lactating period (∼30 days before the expected calving).

All mammary samples were retrieved using a biopsy, which was performed according to the method of with modifications. Briefly, the skin of the selected biopsy site was first shaved and disinfected with ethanol (75%). Then, the site was anesthetized with SU-MIAN-XIN and injected subcutaneously with 1 ml of procaine. A 1.5-cm incision was made in the skin at the midpoint of a rear quarter of the mammary gland and connective tissue using shears and tweezers, which was blunt-dissected away exposing the secretory gland capsule. Mammary tissue biopsy (∼500 mg) was then obtained and immediately placed in liquid nitrogen and subsequently stored at −80°C until RNA isolation. The suture was tied as the cannula was removed and pressure applied to reduce the collection of blood under the skin. Immediately after the experiment, all 12 cows received antibiotic prophylaxis and anti-inflammatory therapy.

### RNA Extraction and Library Preparation for Small RNA Sequencing

Total RNA was extracted using TRIzol reagent (Invitrogen, Carlsbad, CA, United States). Twelve small RNA libraries from RNA integrity and concentration were assessed using the RNA Nano 6000 Assay Kit of the Bioanalyzer 2,100 System (Agilent Technologies, CA, United States). All RNA samples had an RNA integrity number of at least 7.5. Fifteen percent agarose gels separated the total RNA to extract the small RNA (18–30 nt). After precipitation by ethanol and centrifugal enrichment of small RNA samples, the library was prepared according to the method and process of Small RNA Sample Preparation Kit (Illumina, RS-200-0048). The RNA concentration of the library was measured using Qubit^®^ RNA Assay Kit in Qubit^®^ 2.0 for preliminary quantification and then diluted to 1 ng/μl. Insert size was assessed using the Agilent Bioanalyzer 2100 system (Agilent Technologies, CA, United States). After the insert size consistent with expectations, the qualified insert size was accurate quantitative using the Taqman fluorescence probe of AB Step One Plus Real-Time PCR system (Library valid concentration >2 nM). The qualified libraries were sequenced by an Illumina HiSeq 2500 platform and 50-bp single-end reads were generated.

### Identification of Small RNAs

Quality trimming and adaptor removal of the Illumina reads were carried out using Cutadapt 2.8 and Trimmomatic 0.36 (Martin, 2011; Bolger et al., 2014). After filtering for their size (18–30 nt), the cleaned reads were categorized into unique tags and then mapped to the bovine (ARS-UCD 1.2) reference genomes by Bowtie 1.1.1 with one mismatch (Langmead, 2010). All the downstream analyses were based on the mapped small RNA tags.

The matching sequences ranging from 18 to 30 nt were used to align against mirbase 22.0 (http://www.mirbase.org/) to identify known miRNAs by miRDeep2 with a quantifier. pl module (Mackowiak, 2011). The sequences matching other small RNAs, including rRNA, snRNA, repeat RNA, tRNA and snoRNA, were compared with *Bos taurus* noncoding RNA sequences in the Sanger RNA family database (Rfam 12.1) using Infernal 1.1 (Griffiths-Jones et al., 2003; Nawrocki and Eddy, 2013). Unannotated sequences combined with the known miRNA annotation from *Ovis aries*, *Capra hircus*, *Sus scrofa*, *Mus musculus*, and *Homo sapie*ns were used to predict the novel miRNAs according to the characteristic hairpin structure of miRNA precursors by miRDeep2 core module miRDeep2. pl. The miRNAs expressed in at least two samples were considered as novel miRNAs. To make small RNA mapped to unique annotation, we followed the priority rule: known miRNA > rRNA > tRNA > snRNA > snoRNA > repeat > novel miRNA > ta-siRNA.

### Differential Expression Analysis

Differentially expressed (DE) miRNAs between high and low milk protein percentage during peak and non-lactating periods (i.e., HP vs. LP, HD vs. LD) were investigated using the DESeq2 R package (Love et al., 2014). RNA-Seq read counts were modeled by a generalized linear model considering the experimental design, with two phenotypes (high milk protein percentage and low milk protein percentage) and two stages of lactation (peak lactation and non-lactating period). The model for the HP vs. LP and HD vs. LD comparisons only included the phenotype factor. The statistical power of this experimental design was estimated using the SSPA R package (Iterson et al., 2013), which reached 0.63 and 0.76 for the HP vs LP and HD vs. LD, respectively (Supplementary Figure S1). MiRNAs with a *p*-value <0.05 and log_2_ (fold change)| >0.8 were assigned as DE (Zheng et al., 2015). The expression patterns of DE miRNAs across four groups were performed using the k-mean method (Ahmad and Dey, 2007). Using gap statistics, we determined that k = 7 was the optimal choice for distinguishing these miRNAs (Supplementary Figure S2).

### MiRNA Function Prediction and Regulatory Network Construction

We predicted the binding of DE miRNAs to the putative targets using miRanda V3.3a with score ≥50 and energy ≤ −20 kcal/mol (Enright et al., 2003). The predicted target genes were combined with the previous transcriptome profiling data (Li et al., 2016). We identified the correlations between miRNA and target genes in expression using an in-house R script. Briefly, the expressions of miRNA and their targets were sample-matched for all samples. Then for each miRNA, Pearson correlation coefficients were computed for all its targets; only targets significantly (*p*-value <0.05) and inversely correlated with miRNAs in expression were obtained. To evaluate the miRNA-gene regulatory network, GO term and KEGG pathway enrichment analyses were used to investigate putative functions of target genes using DAVID (https://david.ncifcrf.gov/) (Huang et al., 2007). The statistical significance of GO term or KEGG pathway enrichment was measured by Fisher’s exact test with *p*-value <0.1.

### Regulatory Network Construction

We selectively analyzed the DE miRNA-DE mRNA pairs that the targets of DE miRNA were also in DE either HP vs. LP or HD vs. LD. After the functional annotation, miRNAs and their targeting genes and pathways were subjected to the network visualization analysis. The Cytoscape 3.2.1 software was used to construct the network (Smoot et al., 2011).

### The Enrichment Analysis of Genome-Wide Association Studies Signals

We obtained summary statistics of single-trait GWAS for five milk production traits (milk yield, milk protein yield, milk fat yield, milk protein percentage, and milk fat percentage), heifer conception rate, and somatic cell score (SCS) in cattle, as described previously (Jiang et al., 2019). Here we provided a summary. The de-regressed PTAs (predicted transmitting abilities) were used as a phenotype in all seven traits. The imputation phase was from Run 5 of the 1000 Bull Genomes Project (Daetwyler et al., 2014). After sequence marker imputation and quality control, genotypes of 3,148,506 sequence variants for 27,214 Holstein bulls were obtained. The single-trait GWAS analyses were conducted using a mixed-model approach by MMAP (https://mmap.github.io/).

To check whether the SNP effects were more enriched in candidate feature than background regions, we applied a sum-based method for GWAS signal enrichment analysis. The sum-based method uses signals of all markers within a predefined candidate feature. Briefly, we calculated the following summary statistics for candidate feature:

$$T_{sum}=\overset{m_{g}}{\underset{i=1}{\sum }}{\text{β}}^{2}$$ (1)

in which $T_{sum}$ is the summary statistics for a tested feature group, is the number of SNPs located in the candidate feature and β is the estimate of marker effect obtained from the GWAS summary statistics. Using Eq. 1, we calculated the $T_{sum}$ for candidate feature.

To perform the permutation test in background feature, we first assumed the SNP markers of the GWAS summary statistics located in background regions which were numbered as 1 … *M*. The observed SNPs located in the prior feature were $N_{1}$ , $N_{2}$ , $N_{3}$ , …, $\;N_{n}$ . Their test statistics were ${\text{β}}_{N_{1}}^{2}$ , $\;{\text{β}}_{N_{2}}^{2}$ , ${\text{β}}_{N_{3}}^{2}$ , …, ${\text{β}}_{N_{n}}^{2}$ . We chose number R within 1 ∼ M. Then, the observed SNP set was shifted to the new rank order ( $P_{1}$ , $P_{2}$ , $P_{3}$ , …, $P_{n}$ ) based on random number R using the following formula:

$$P_{i}=\{\begin{array}{c} N_{i}+R,\;\;\;\;\;\;\;\;\;\;\;\;N_{i}+R\leq M\\ N_{i}+R-M\;,\;\;N_{i}+R>M \end{array}$$ (2)

All test statistics were moved to the new positions, with the remaining markers maintaining the original order. A new summary statistic of a background region ( ${\text{β}}_{P_{1}}^{2}$ , $\;{\text{β}}_{P_{2}}^{2}$ , $\;{\text{β}}_{P_{3}}^{2}$ , …, ${\text{β}}_{P_{n}}^{2}$ ) was calculated based on the original position of the feature. The permutation in background regions was repeated 10,000 times for each studied candidate feature and an empirical *p*-value was then calculated based on one-tailed tests of the proportion of randomly sampled summary statistics larger than that observed using the following formula:

$$P=\left(N_{over}\mathrm{+1}\right)/10001$$ (3)

where $N_{over}$ represents the times of the permutated $T_{sum}$ which was found larger than that of the candidate feature $T_{sum}$ . We corrected empirical *p* values for the multiple testing using the FDR method implemented in R (p.adjust function) and then considered FDR < 0.05 as significant. To avoid bias by the *DGAT1* gene, we conducted the GWAS enrichment analysis by excluding SNPs located in the *DGAT1* gene or 1-Mb upstream/downstream extended region of the *DGAT1* gene. This sum-based method for GWAS signal enrichment analyses using Perl scripts is available (https://github.com/WentaoCai/GWAS_enrichment).

## Results

### Overview Over Small RNA Sequencing

**FIGURE 1:**
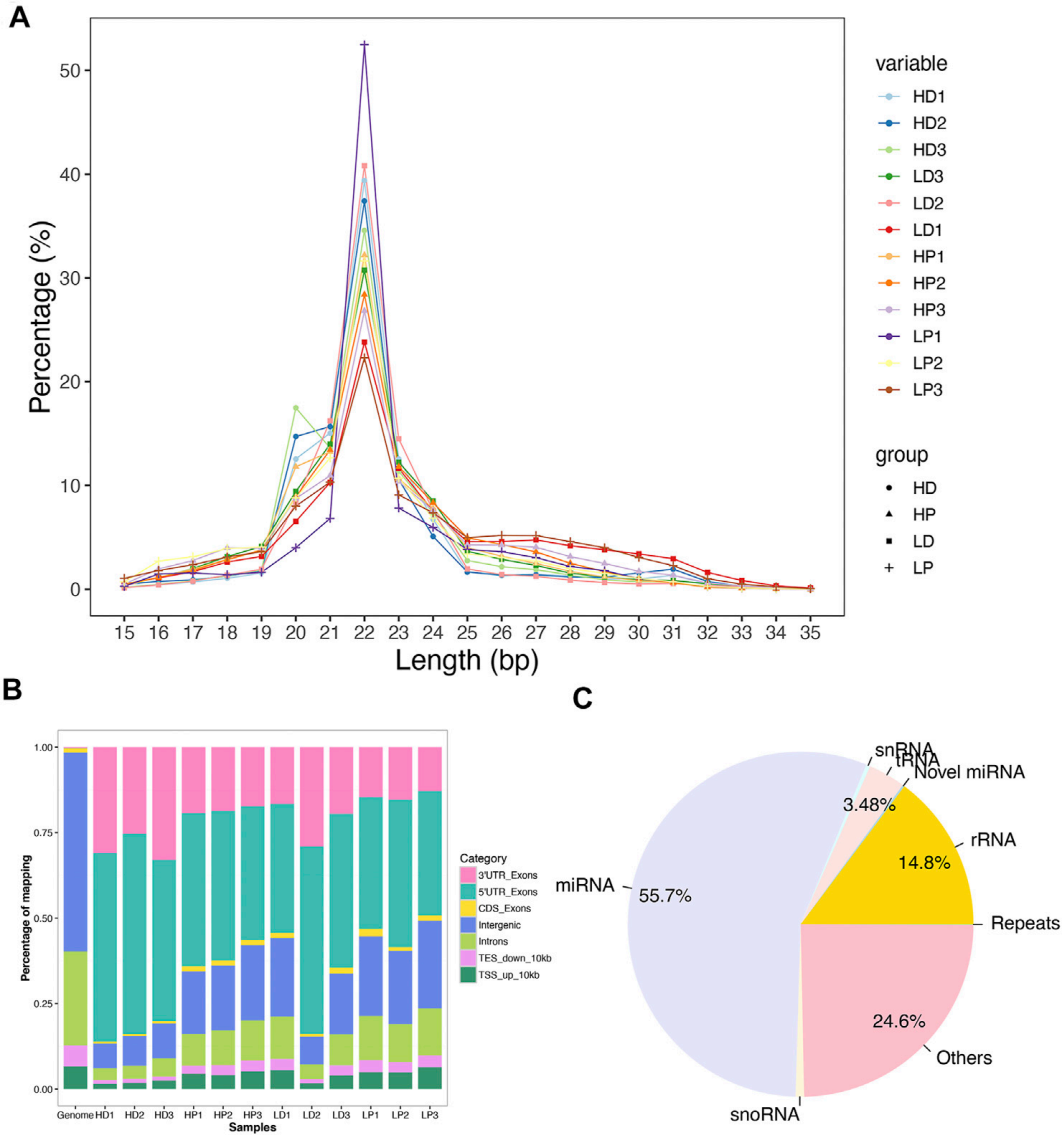
Mapping statistics. **(A)** Length distribution of the mapped reads across 12 libraries. **(B)** The genome distributions of the mapped reads for all 12 samples. **(C)** The relative abundance of different classes of small RNAs in the total reads was successfully mapped to the bovine genome.

To study miRNAs in milk protein synthesis’ complex process, we profiled miRNA expression differences between the high milk protein percentage and low milk protein percentage groups in both lactation and non-lactating period using small RNA sequencing. After trimming adaptor sequences and removing contaminated reads, an average of 23.0 million clean reads ranges from 22.4 to 23.9 million were generated. Then, we categorized them into unique tags; an average of 1.1 million unique tags was obtained (Supplementary Table S2). We separately mapped clean reads and unique tags to the bovine (ARS-UCD1.2) reference genomes. The mapping rates were about 90.0 and 74.4% using total clean reads and unique tags, respectively (Supplementary Table S3). The majority of the mapped reads ranged from 21 to 23 nt in length and the 22-nt small RNA was the most abundant (Figure 1A). As expected, most reads were observed to match with the 3′-untranslated region (UTR) and 5′-UTR allocating miRNAs (Figure 1B). These results confirm that the small RNA sequencing process is reliable in our study. The residual fraction of mapped reads not corresponding to miRNAs was distributed among a miscellanea of annotated regions, including rRNAs (14.76%), tRNAs (3.48%), snoRNAs (0.38%), snRNAs (0.78%) and repeats (0.04%) (Figure 1C).

### Identification of Known and Novel miRNAs

**FIGURE 2:**
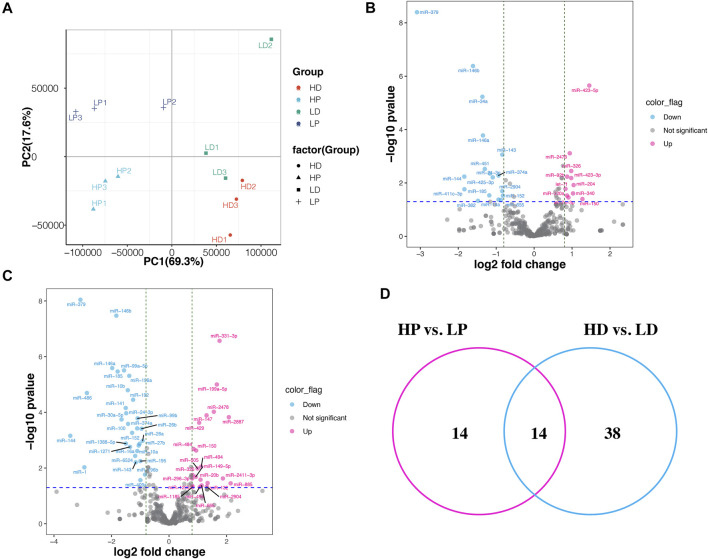
Differentially expressed miRNAs between high and low milk protein percentage. **(A)** The principal component analysis (PCA) scatter plot of miRNA expression in the 12 samples. PCA plot showing variance of the three biological replicates of each of the groups. The percentages on each axis represent the percentages of variation explained by the principal components. **(B)** Volcano plot displaying differentially expressed miRNAs of HP vs. LP. The pink and blue dots represent the significantly upregulated and down-regulated miRNA; the gray dots represent miRNAs whose expression levels do not reach statistical significance. **(C)** Volcano plot displaying differentially expressed miRNAs of HD vs. LD. **(D)** Venn diagram depicting commonly and uniquely DE miRNAs detected by HP vs. LP and HD vs. LD.

We identified 388 known miRNAs expressed in at least two samples, which accounted for 38.7% of all known bovine miRNAs deposited in miRbase 22.0 (transcripts per million, TPM >0.5). Despite differences in sample characteristics, samples from the same group clustered together based on their miRNA expression profiles (Figure 2A). The first principal component (PC1), explaining the most variance in miRNA expression, separates the samples by lactating stage. PC2 separates the samples by the phenotype of milk protein percentage. We also compared the miRNAs with the top greatest expression (top 20) in the mammary tissue at lactation and non-lactating periods (Supplementary Figure S3). The top expressed miRNAs in both of the two stages were the same except for miR-142 and miR-126, which were explicitly expressed higher in lactation and non-lactating period, respectively. The highest expression of miRNAs in lactation was miR-148a, while miR-143 was the most highly expressed in the non-lactating period. In addition, we characterized 297 novel miRNAs expressed in at least two samples, including 172 and 188 novel miRNAs identified in lactation and non-lactating period. Interestingly, we found 17 novel miRNAs expressed in all 12 samples (Supplementary Table S4).

### Differentially Expressed miRNA Within Extreme Phenotypes in Lactation and Non-lactating Period

**FIGURE 3:**
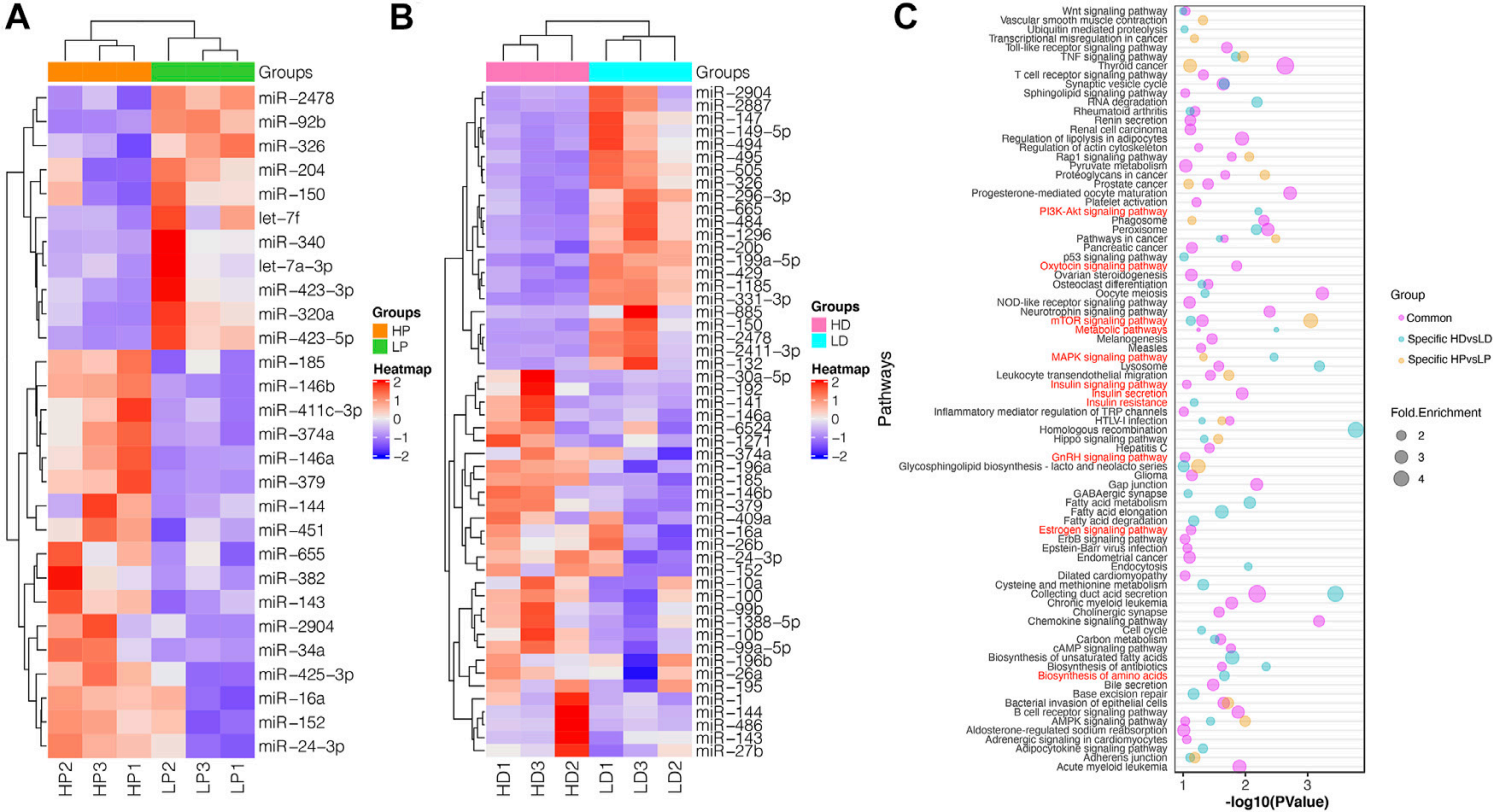
The functional annotation of DE miRNAs. **(A,B)** Cluster analysis of DE miRNAs in HP vs. LP and HD vs. LD based on their standardized expression by z-score. Red indicates higher expression and blue shows lower expression. **(C)** Predominant function categories targeted by common DE miRNAs, lactation-specific DE miRNAs and non-lactating-specific DE miRNAs. More significant values and shapes were suggesting higher relevance and higher enriched fold, respectively. The red labels represent the milk protein-associated pathways.

We identified 28 DE miRNAs between HP and LP groups in lactation, including 11 upregulated and 17 downregulated miRNAs in the LP group relative to the HP group (*p*-value <0.05, |log_2_ (fold change)| >0.8, as shown in Figures 2B,C; Supplementary Table S5A). A total of 52 miRNAs were DE between the HD and LD groups in the non-lactating period, including 22 upregulated and 30 downregulated miRNAs in the LD group relative to the HD group (Supplementary Table S5B). Interestingly, we found that 14 DE miRNAs exhibited common expression level differences across the two comparison groups (Table 1; Figure 2D). The clustering heat map of all 66 DE miRNA expression profiles from HP vs. LP or HD vs. LD is shown in Figure 3A.

**TABLE 1:** The differentially expressed miRNAs between high and low milk protein content in lactation and non-lactation.

Group	DE miRNAs number	DE miRNA list
HP vs LP	28	Up: let-7a-3p, let-7f, miR-150, miR-204, miR-2478, miR-320a, miR-326, miR-340, miR-423-3p, miR-423-5p, miR-92b
		Down: miR-143, miR-144, miR-146a, miR-146b, miR-152, miR-16a, miR-185, miR-24–3p, miR-2904, miR-34a, miR-374a, miR-379, miR-382, miR-411c-3p, miR-425–3p, miR-451, miR-655
HD vs LD	52	Up: miR-1185, miR-1296, miR-132, miR-147, miR-149-5p, miR-150, miR-199a-5p, miR-20b, miR-2411-3p, miR-2478, miR-2887, miR-2904, miR-296-3p, miR-326, miR-331-3p, miR-429, miR-484, miR-494, miR-495, miR-505, miR-665, miR-885
		Down: miR-1, miR-100, miR-10a, miR-10b, miR-1271, miR-1388–5p, miR-141, miR-143, miR-144, miR-146a, miR-146b, miR-152, miR-16a, miR-185, miR-192, miR-195, miR-196a, miR-196b, miR-24–3p, miR-26a, miR-26b, miR-27b, miR-30a-5p, miR-374a, miR-379, miR-409a, miR-486, miR-6524, miR-99a-5p, miR-99b
Common	14	miR-143, miR-144, miR-146a, miR-146b, miR-150, miR-152, miR-16a, miR-185, miR-24–3p, miR-2478, miR-2904, miR-326, miR-374a, miR-379

### Target Gene Prediction of Differentially Expressed miRNAs

To better understand the function of DE miRNAs, putative target genes were predicted using the 3′-UTR sequence of mRNA by the miRanda software. We predicted 9,156 target mRNAs for the 28 DE miRNAs in HP vs LP and 10,045 target mRNAs for the 52 DE miRNAs in HD vs. LD. To identify target genes with high confidence, we performed correlation analysis between DE miRNAs and their target genes in the expression levels in the 12 mammary samples. The expression of target genes from the 12 similar samples was quantified by RNA-seq mentioned in our previous study (Li et al., 2016). We detected 28 DE miRNAs inversely correlated with 1,685 targets resulting in 2,468 miRNA-mRNA pairs for HP vs. LP and 52 DE miRNAs inversely correlated with 2,280 targets resulting in 3,697 miRNA-mRNA pairs for HD vs. LD (Supplementary Tables S6A,B). For the 14 common DE miRNAs across HP vs. LP and HD vs. LD, we found 914 inversely correlated targets resulting in 1,210 miRNA-mRNA pairs (*p*-value <0.05, Supplementary Table S6C).

### Functional Annotation of Differentially Expressed miRNAs

To functionally classify the DE miRNAs, GO and KEGG enrichment analyses were performed for DE miRNAs’ confident target genes in lactation and non-lactating period, respectively. Functional annotation showed that these 914 target genes of common DE miRNAs were significantly enriched in 60 pathways and 123 GO terms. Many pathways were associated with protein synthesis, insulin secretion, mTOR signaling pathway, estrogen signaling pathway, insulin signaling pathway and GnRH signaling pathway. Many GO terms were involved in protein synthesis, such as protein transport, *trans*-Golgi network, metabolic process and protein serine/threonine kinase activity (Supplementary Table S7A).

For specifically DE miRNAs in lactation, their target genes were enriched in 18 pathways and 110 GO terms. The pathways were involved in the mTOR signaling pathway, TNF signaling pathway, leukocyte transendothelial migration and MAPK signaling pathway. The functional terms involved in protein synthesis were noticed for positive regulation of transcription, post-Golgi vesicle-mediated transport, mRNA 3′-UTR binding and ER to Golgi transport vesicle (Supplementary Table S7B). For specifically DE miRNAs in the non-lactating period, their target genes were enriched in 37 pathways and 205 GO terms. Several target genes were observed to be involved in the PI3K-Akt signaling pathway, metabolic pathways and mTOR signaling pathway. Their functional terms were associated with protein transport, transcription, vasculogenesis and positive regulation of gene silencing by miRNA (Supplementary Table S7C). All enriched KEGG pathways and the top 10 significant GO terms are shown in Figure 3B.

**FIGURE 4:**
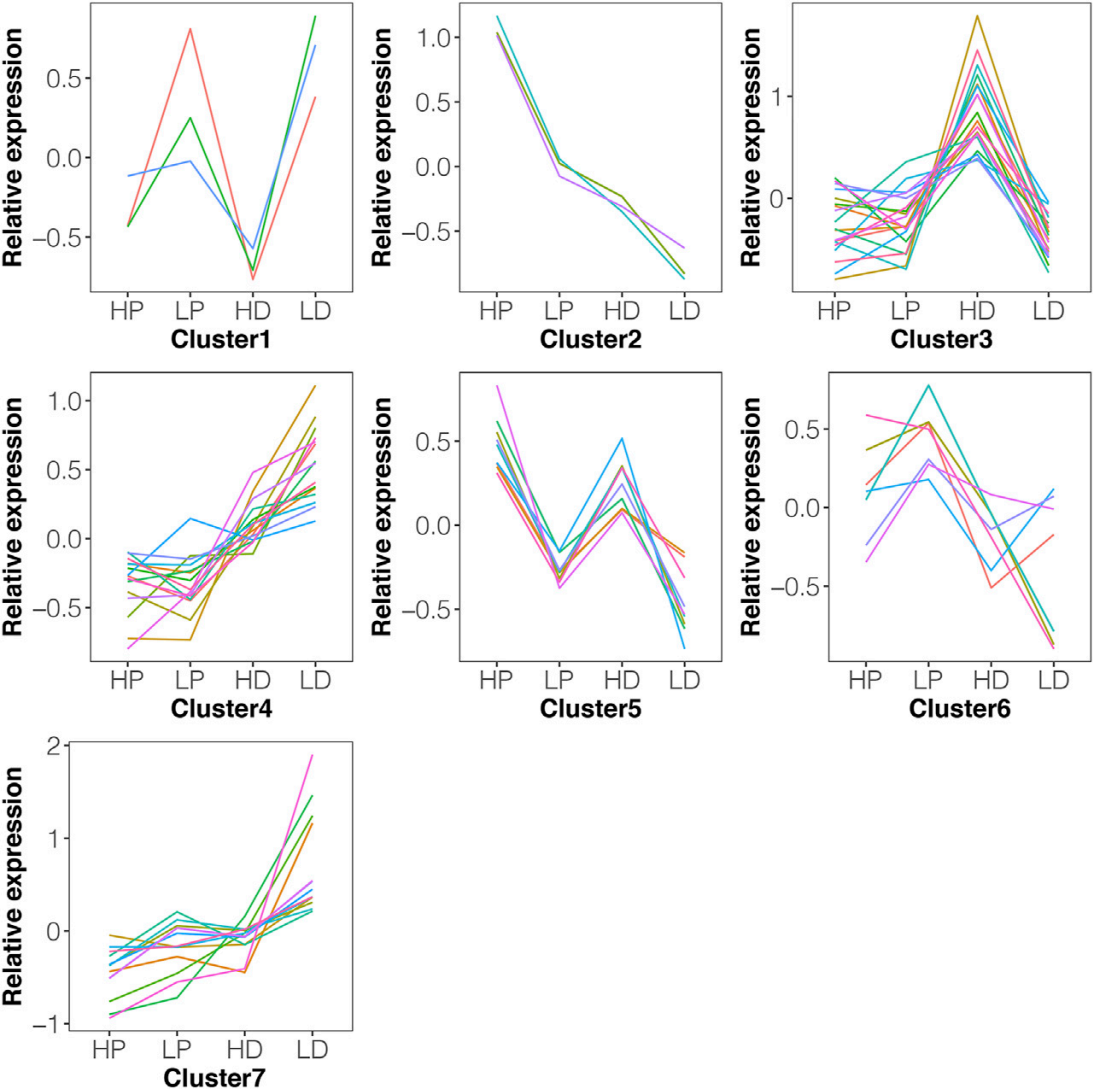
The expression pattern of DE miRNAs using k-means clustering. The 66 DE miRNAs in either HP vs. LP or HD vs. LD could be divided into seven distinct clusters with differentially expression level pattern changes. The *y*-axis represents the relative expression using mean normalization. Most clusters (such as clusters 1, 2, 5 and 7) revealed that the expression change pattern of miRNAs in HP vs. LP was similar to HD vs. LD.

Trends in DE miRNAs in lactation or non-lactating period were examined using k-means clustering, which revealed that 66 DE miRNAs in either HP vs. LP or HD vs. LD could be divided into seven distinct clusters with differentially expression level pattern changes (Figure 4). Most clusters (such as clusters 1, 2, 5 and 7) revealed that the expression change pattern of miRNAs in HP vs LP was similar to HD vs LD. Functional annotation reveals that these 26 miRNAs with similar expression patterns were involved in a variety of biological processes, such as biosynthesis of antibiotics, metabolic pathways and TNF signaling pathway (Supplementary Table S8). Clusters 3 and 4 indicated that some miRNAs were stably expressed between HP and LP, while they were dynamically changed between HD and LD.

### Regulatory Networks for Differentially miRNAs-mRNAs

**FIGURE 5:**
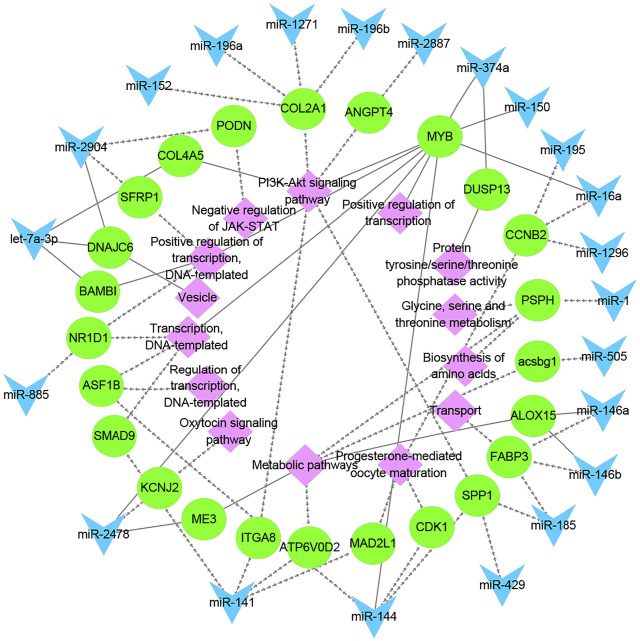
The functional network of candidate miRNAs, mRNAs, and pathways. The blue triangles, green circles, and pink diamonds represent miRNAs, mRNAs and pathways, respectively. The dashed and solid lines represent the lactating and non-lactating period networks, respectively.

To better understand the relationship between miRNAs and milk protein traits, we selectively analyzed the 214 miRNA-mRNA pairs. Both miRNAs and their targets were DE in HP vs LP or HD vs LD. We found that 22 DE miRNAs potentially regulated 24 DEGs which were involved in milk protein synthesis (Table 2). For example, *PSPH*, as a phosphoserine phosphatase that functions as phosphotransferase that is responsible for the third and last steps in l-serine formation, was involved in the biosynthesis of amino acids, metabolic pathways and glycine, serine and threonine metabolism. The expression of miR-1 was negatively correlated with *PSPH*. *FABP3*, targeted by miR-146b and miR-185, influences fat and protein content in cattle (Kulig et al., 2013). Additional genes are listed in Table 2. The networks of candidate target genes involved in milk protein synthesis through various pathways are shown in Figure 5.

**TABLE 2:** The differentially expressed miRNAs with their potential target genes related to milk protein synthesis.

Group	miRNAs	log2 (fold change)	*p* value	padj	Targets
HP vs LP	let-7a-3p	0.90	0.0351	0.2489	BAMBI, COL4A5, DNAJC6
	miR-150	1.27	0.0401	0.2684	MYB
	miR-144	−1.84	0.0058	0.0985	MYB
	miR-16a	−0.94	0.0422	0.2706	MYB
	miR-2478	0.94	0.0008	0.0351	MYB
	miR-146a	−1.35	0.0002	0.0094	ALOX15, FABP3
	miR-146b	−1.61	4.15E-07	0.0001	ALOX15, FABP3
	miR-2478	0.94	0.0008	0.0351	ME3, MYB
	miR-2904	−0.84	0.0203	0.1910	DNAJC6
	miR-374a	−0.94	0.0053	0.0985	MYB, DUSP13
HD vs LD	miR-1	−2.94	0.0094	NA	PSPH
	miR-1296	0.81	0.0443	0.1589	CCNB2
	miR-141	−1.51	0.0001	0.0012	SMAD9, ITGA8, ATP6V0D2, MAD2L1
	miR-195	−1.00	0.0056	0.0329	CCNB2
	miR-16a	−1.05	0.0016	0.0113	CCNB2
	miR-1271	−1.36	0.0017	0.0123	COL2A1
	miR-152	−1.28	0.0005	0.0048	COL2A1
	miR-196b	−0.83	0.0172	0.0808	COL2A1
	miR-196a	−1.38	4.89E-06	0.0001	COL2A1
	miR-2887	2.08	0.0001	0.0018	ANGPT4
	miR-429	1.05	0.0002	0.0025	SPP1
	miR-505	0.99	0.0104	0.0529	ACSBG1
	miR-885	2.14	0.0355	NA	NR1D1
	miR-144	−3.43	0.0007	0.0060	ASF1B, SPP1, CDK1
	miR-146a	−1.97	0.0000	0.0001	FABP3
	miR-146b	−1.83	3.37E-08	4.04E-06	FABP4
	miR-185	−1.79	3.42E-06	0.0001	SPP1, FABP3
	miR-2478	1.56	0.0001	0.0014	KCNJ2
	miR-2904	1.31	0.0470	0.1612	PODN, SFRP1

### Differentially Expressed miRNA Genes are Enriched With GWAS Signals of Milk Protein Traits

To assess whether DE miRNAs were associated with GWAS signals, we applied enrichment analysis from GWAS for all correlated targets of DE miRNAs across five milk traits (milk yield, milk protein yield, milk fat yield, milk protein percentage and milk fat percentage), one reproduction trait (heifer conception rate) and one health trait (somatic cell sore, SCS). Since very few imputed SNPs were observed within the miRNA precursor regions due to their short lengths, the analysis included the flanking ±50 kb sequences of DE miRNA precursors to capture proximal SNPs in the regulatory regions. The background regions were all miRNA precursors in miRbase 22.0 and their flanking ±50-kb regions. We did not detect significant enrichments for milk production in both HP vs LP and HD vs LD (Supplementary Table S9a). However, after removing 1,737 SNPs close to *DGAT1*, significant (FDR < 0.05) enrichments were observed for the milk protein percentage trait in DE miRNA of HP vs LP, while the GWAS signals of the protein trait were enriched in DE miRNAs of HD vs LD (FDR < 0.1, Table 3). Next, the DE miRNAs were separated into upregulated and downregulated miRNAs based on their log2FC >0 (up) or <0 (down) across comparison groups. The milk protein GWAS signals were significantly more enriched in upregulated miRNAs than in downregulated miRNAs in HP vs LP, while the opposite results were found in HD vs LD (Supplementary Table S9B). These results suggested that the milk protein variations in the traits may be associated with the DE miRNA genes.

**TABLE 3:** The enrichment results of GWAS signals for the DE miRNA precursors and targets of DE miRNAs.

Traits	DE miRNA precursors				Targets of DE miRNAs			
	HP vs. LP		HD vs. LD		HP vs. LP		HD vs. LD	
	*p*-value	FDR	*p*-value	FDR	*p*-value	FDR	*p*-value	FDR
Milk	0.149	0.260	0.191	0.268	0.007	0.024*	0.066	0.093
Fat	0.244	0.342	0.143	0.250	0.026	0.045*	0.063	0.093
Fat percentage	0.086	0.200	0.139	0.250	0.013	0.030*	0.199	0.232
Protein	0.064	0.200	0.011	0.080	0.002	0.014*	0.002	0.014*
Protein percentage	0.005	0.033*	0.289	0.289	0.043	0.060	0.061	0.093
SCS	0.443	0.516	0.110	0.250	0.076	0.089	0.004	0.019*
Heifer_Conc_Rate	0.649	0.649	0.244	0.285	0.584	0.584	0.642	0.642

The 1,737 SNPs close to DGAT1 genes were discarded in GWAS enrichment analysis. The significant (<0.05) false discovery rate (FDR) correction of p-value were marked with star symbols and bold values.

### Target Genes of Differentially Expressed miRNAs are Enriched With GWAS Signals of Milk Protein Traits

To investigate the joint effect of genetic variations in miRNA targets on milk production traits in dairy cattle, we conducted the GWAS enrichment analysis using inversely and significantly correlated targets of DE miRNA. Only SNPs located in targets or in the 5 kb extended region of targets were included in calculating the squares of their effects. For comparison, 10,000 random SNP sets located in all annotated genes (Ensemble 95) or their 5 kb extended areas were generated. As shown in Figure 5A, the correlated targets of DE miRNAs were enriched with GWAS signals of the milk protein trait (FDR < 0.05, Supplementary Table S10A). After correcting the DGAT1 bias, significant (FDR < 0.05) enrichments were observed for all five milk production traits in HP vs LP. For the targets of DE miRNAs in HD vs LD, significant enrichments were kept for milk protein and SCS traits. Similar with the GWAS enrichment analysis of DE miRNA genes, we also found that the milk protein GWAS signals were significantly more enriched in targets of upregulated miRNAs than in targets of downregulated miRNAs in HP vs LP, while the opposite results were found in HD vs LD (Supplementary Table S10B). In addition, we did not find any enrichments in either HP vs LP or HD vs LD for the GWAS signals of the heifer conception rate trait (Supplementary Table S10B).

## Discussion

In this study, we obtained a comprehensive landscape of miRNAs associated with milk protein in the context of miRNA profiles across 12 mammary tissue samples during two different stages of lactation. Importantly, we identified candidate miRNAs and networks related to milk protein by integrating miRNAs, transcriptome and GWAS signals. These findings provide valuable insights for lactogenesis and yield a suite of molecular breeding resources to optimize the content of milk proteins.

There were 388 known miRNAs expressed in our study, accounting for 38.7% of all known bovine miRNAs deposited in miRbase 22.0. A total of 297 novel miRNAs were detected in this study, which will considerably increase bovine miRNAs’ repertoire. A weakness of this study is the lack of visual inspection for the biopsy sample, but we found that the top expressed miRNAs of our mammary biopsy sample were similar with those of previous studies (Jin et al., 2014; Billa et al., 2019). The differentially regulated expression patterns of miRNAs in mammary gland tissue underscore that the synthesis and secretion of milk protein involve a high level of posttranscriptional regulation of gene expression by miRNAs. The 14 DE miRNAs between high and low milk protein percentages across both lactation and non-lactating periods suggest that these miRNAs may partially regulate the functions of the same biological or physiological processes in the two periods.

After combining the target prediction with expression correlation analysis, we matched 1,685 inversely correlated targets that resulted in 2,468 miRNA-mRNA pairs for HP vs LP and 2,280 inversely correlated targets that resulted in 3,697 miRNA-mRNA pairs for HD vs LD. Functional annotation showed that these inversely correlated targets of common DE miRNAs across two stages were associated with mTOR signaling pathway, estrogen signaling pathway, insulin signaling pathway, and GnRH signaling pathway, implying that these miRNAs could be critical to factors that affect milk quality and yield. It should be noted that some of the common DE miRNAs in this study have been previously suggested to play essential roles in milk protein synthesis. For example, miR-152 negatively regulates DNA methyltransferase 1 (*DNMT1*), decreasing the global DNA methylation and increasing the expression of serine/threonine protein kinase Akt (*AKT*) and peroxisome proliferator-activated receptor gamma (*PPARγ*) (Wang et al., 2014). These target genes of DE miRNAs, specifically for lactation, were involved in positive transcription, mRNA 3′-UTR binding, and ER to the Golgi transport vesicle. Also, miR-423-5p has been shown to regulate AMPK-gamma1 (*AMPKγ1*) negatively. The 3′-UTR SNP of *AMPKγ1* was influential on the milk and protein yield traits. This mutation also changed target mRNA base-pairing to miR-423-5p, which implied that miR-423-5p plays an important role in milk metabolism pathways (Mahmoudi et al., 2015). These target genes of DE miRNAs, especially for the non-lactating period, were also associated with some milk protein metabolisms, such as PI3K-Akt signaling pathway, metabolic pathways and mTOR signaling pathway. For example, miR-486 directly downregulates *PTEN* gene expression, altering the expression of downstream genes, such as *AKT* and *mTOR*. miR-486 as a downstream regulator of *PTEN* is required for the development of the cow mammary gland (Li et al., 2015a).

The DE miRNA-DEG regulatory networks provided a comprehensive profile for understanding the mechanism of milk protein synthesis in cows. Twenty-two DE miRNAs which potentially regulated 24 DEGs associated with milk protein metabolism were identified. MiR-1 is a known suppressor involved in PI3K-AKT, mTOR, and NFκB pathways (Safa et al., 2020). miR-1 controls cholesterol synthesis and regulates mammary proliferation by targeting *IGF1* and *TBX3* in the sow’s mammary gland (Lin et al., 2020). Here, we found that the expression of miR-1 was negatively correlated with *PSPH*, which is an insulin-responsive gene in bovine mammary that is involved in protein synthesis (Menzies et al., 2009). Besides, proteins encoded by *PSPH* are engaged in serine synthesis (Brearley et al., 2019; Xuan et al., 2020). miR-146b was upregulated in the mammary glands of the HP group, which was reported to be involved mainly in leukemia, epidermal growth factor receptor (*EGFR*) signaling, MAPK, and nuclear factor kappa-light-chain-enhancer of activated B cells (NF-κB) signaling pathways (Mathews et al., 2004; Taganov et al., 2006; Xiang et al., 2014). Moreover, miR-146b was associated with mammary gland development and stem cell activity (Wicik et al., 2016). The expression of *FABP3* was negatively correlated with miR-146b. SNPs within *FABP3* have been reported to influence fat and protein content in cattle (Kulig et al., 2013). These findings indicated that the expression change in DEGs and DE miRNAs within networks might contribute to milk protein metabolism in cows.

We integrated DE miRNA genes and their correlated targets with GWAS signals of milk production traits using the sum-based marker-set test method, which has been demonstrated to have higher power or at least equal to most commonly used marker-set test methods in polygenic traits (Sorensen et al., 2017; Fang et al., 2018). Our analysis revealed significant enrichment of milk protein GWAS signals in proximity to the precursors and target genes of DE miRNAs, especially to DE miRNAs in lactation, which implied that the DE miRNAs of lactation were more associated with milk protein traits. The GWAS signals of heifer conception rate trait were not enriched in targets of milk protein-associated miRNAs, which could be explained by negative genetic correlations between milk production traits and reproduction traits (Strucken et al., 2012; Tiezzi et al., 2012). Previous studies have reported that the DEGs in non-lactating periods could help the mammary tissue prevent issues with inflammation and udder disorders (Li et al., 2016). Of interest, we found that the DE miRNAs of the non-lactating period were related to the SCS trait. The differences in enrichments of up/downregulated miRNAs between lactation and non-lactating period indicated that the miRNAs might have different patterns of regulation involved in milk-related activities.

## Conclusion

This study integrated small RNA sequencing with RNA-seq in the mammary gland to detect genes/pathways associated with milk protein synthesis in cows. We provide genomic evidence that DE miRNA genes in lactation are significantly enriched with GWAS signals for milk protein percentage traits and that enrichments within DE miRNA targets are significantly higher than in random gene sets for the majority of milk production traits. Responsive miRNAs in the mammary gland played roles in the regulation of the milk protein synthesis and the dysregulation of overall metabolism, providing novel milk-biological insights into the genetic mechanisms. The results should further enhance our understanding of miRNA expression profiles associated with milk protein concentration, allowing us to develop more effective breeding strategies.

## Data Availability

The datasets presented in this study can be found in online repositories. The names of the repository/repositories and accession number(s) can be found below: https://www.ncbi.nlm.nih.gov/, PRJNA689373, https://www.ncbi.nlm.nih.gov/geo/, GSM5511040.

## Supplementary Material

Supplementary Figure 1 The statistical power of this experimental design. The points with green and red color represent the statistical power for HP vs LP and HD vs LD, respectively. When sample size is 3, the statistical power reached 0.63 and 0.76 for the HP vs. LP and HD vs. LD, respectively.

Supplementary Figure 2 The optimization of the k-mean using gap statistics. We determined that *k* = 7 was the optimal choice for distinguishing these DE miRNAs.

Supplementary Figure 3 Expression of the top 20 highest miRNAs in lactation and non-lactation period.

Supplementary Figure 4 The enrichment of GWAS signals for up-regulated and down-regulated DE miRNAs in HP vs. LP and HD vs. LD. **(A)** The GWAS enrichment for DE miRNA precursors. The dark green and gold color represent the *p*-values of permutation tests for the up-regulated and down-regulated DE miRNAs, respectively. **(B)** The GWAS enrichment for targets of DE miRNAs.

## Abbreviations

DE, differentially expressed; DEG, differentially expressed genes; DGAT1, diacylglycerol O-acyltransferase 1; DHI, dairy herd improvement system; EGFR, epidermal growth factor receptor; FABP3, fatty acid binding protein 3; GO, gene ontology; GWAS, genome-wide association study; HD, high milk protein percentage in dry (non-lactating) period; HP, high milk protein percentage in peak period (lactation); IGF1, insulin-like growth factor 1; KEGG, Kyoto Encyclopedia of Genes and Genomes; LD, low milk protein percentage in dry (non-lactating) period; LP, low milk protein percentage in peak period (lactation); MAPK, A mitogen-activated protein kinase; mTOR, mammalian target of rapamycin; NGS, next-generation sequencing; PCA, principal component analysis; PSPH, phosphoserine phosphatase; PTAs, predicted transmitting abilities; PTEN, phosphatase and tensin homolog; QTL, quantitative trait locus; RISC, RNA-induced silencing complex; SCS, somatic cell sore; SNP, single-nucleotide polymorphism; TBX3, T-box transcription factor 3; TNF, tumor necrosis factor; TPM, transcripts per million; UTR, untranslated region.
