# Supplementary figures and images for: Integrated Small RNA Sequencing, Transcriptome and GWAS Data Reveal microRNA Regulation in Response to Milk Protein Traits in Chinese Holstein Cattle
Source: Front Genet. 2021 Oct 12;12:726706. doi: 10.3389/fgene.2021.726706 (PMC8546187; doi:10.3389/fgene.2021.726706)

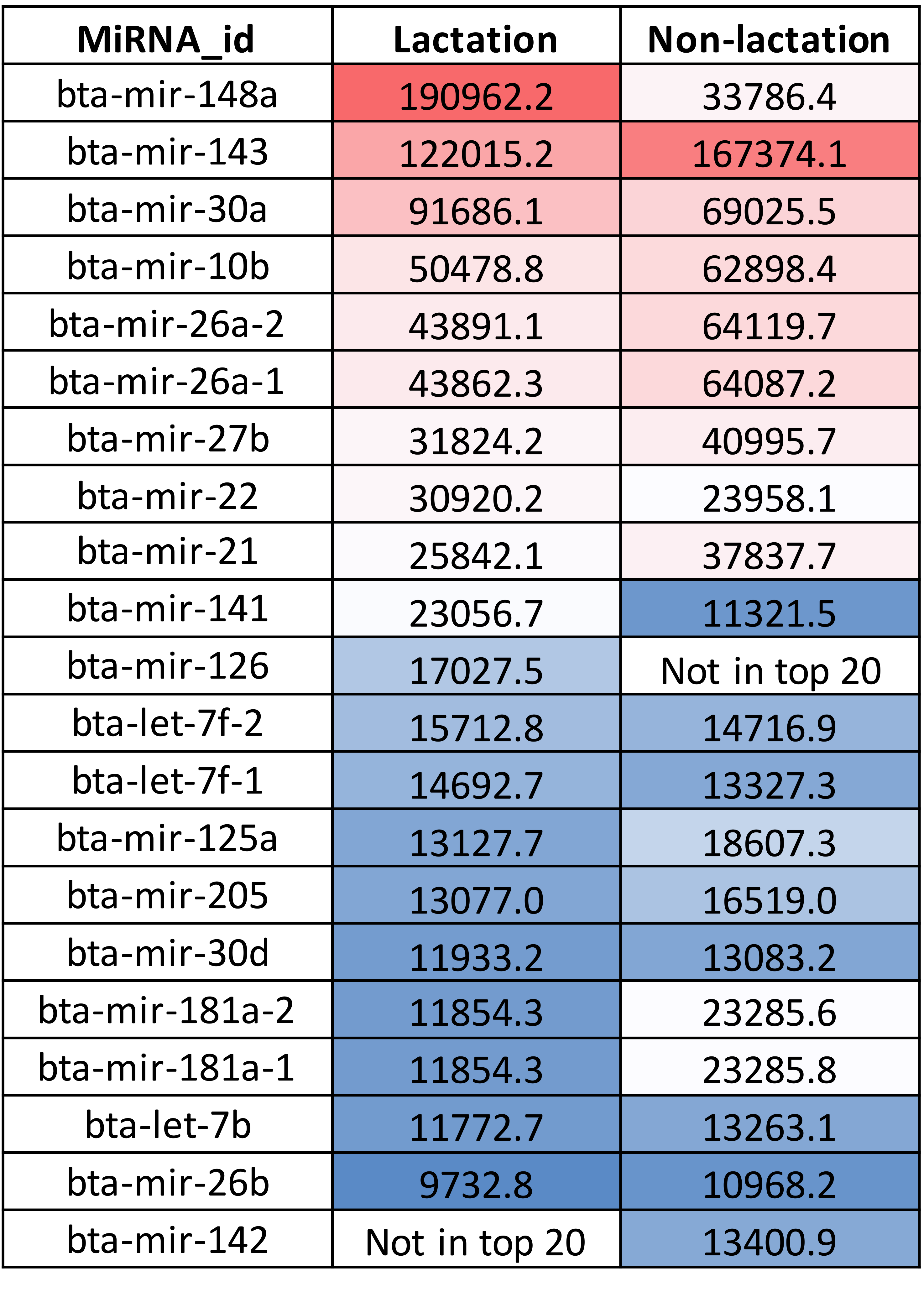

Supplement: Supplementary file 4 [file Image3.TIF]

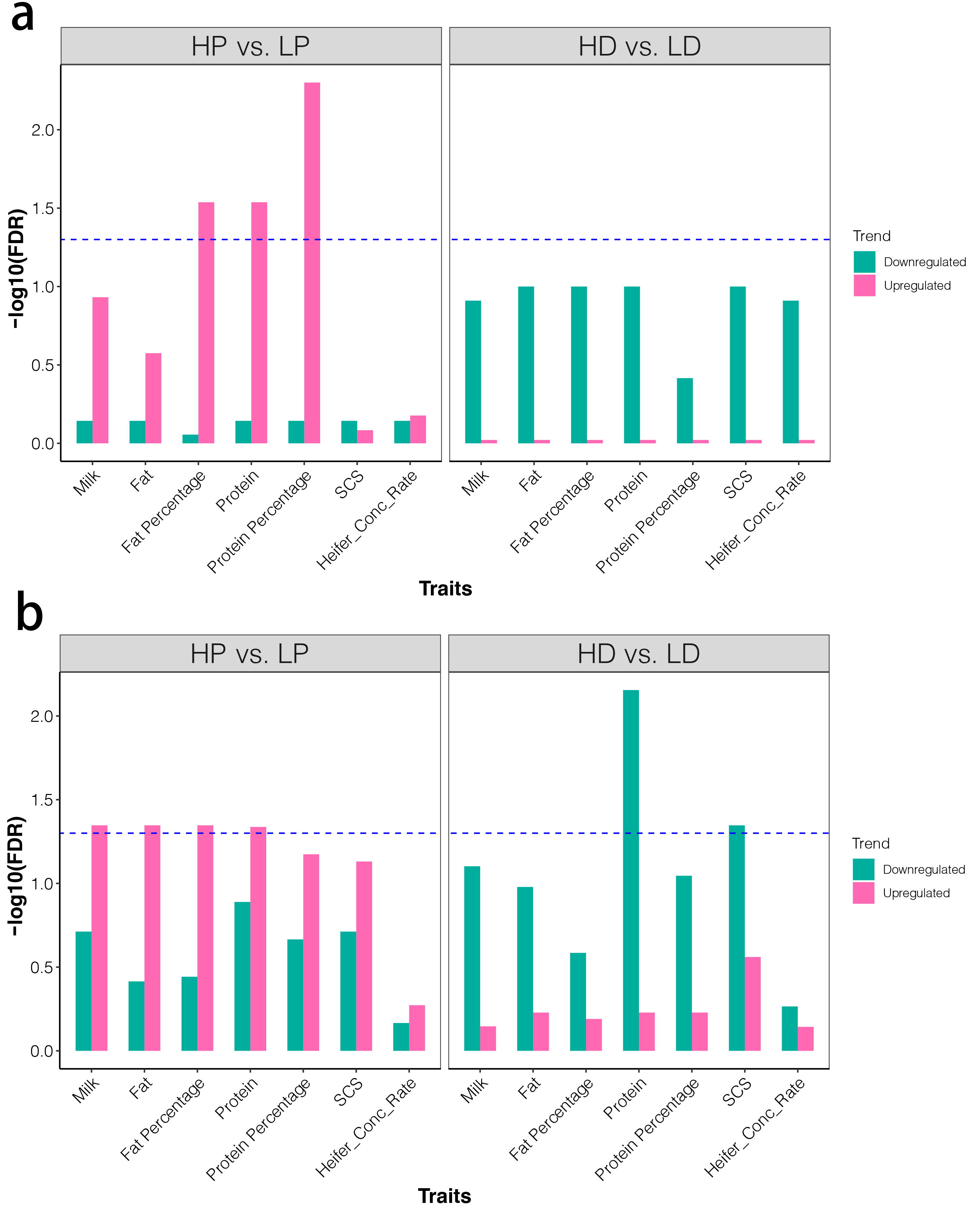

Supplement: Supplementary file 5 [file Image4.TIF]

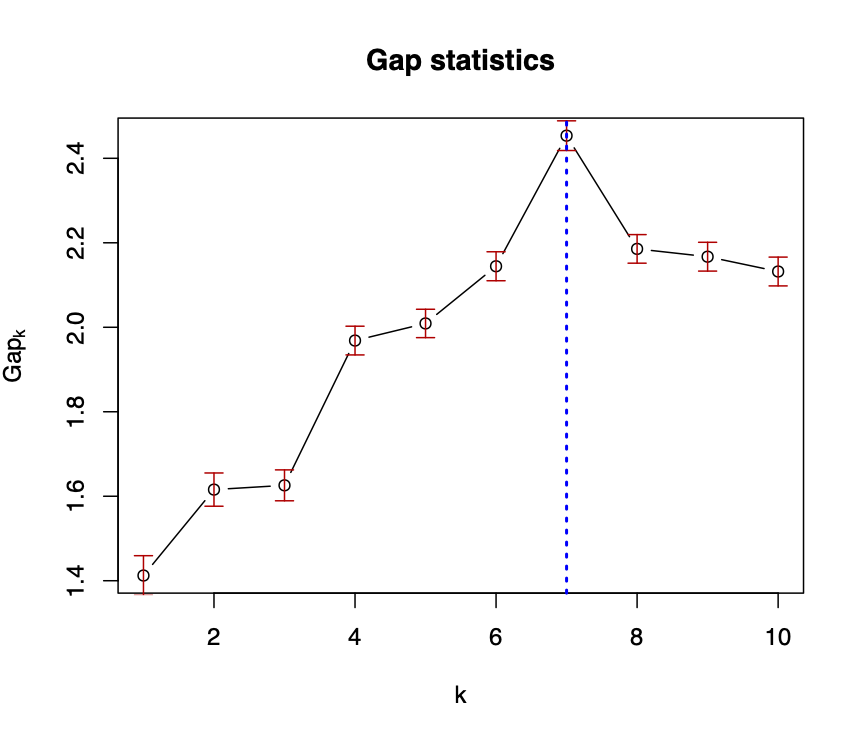

Supplement: Supplementary file 6 [file Image2.TIF]

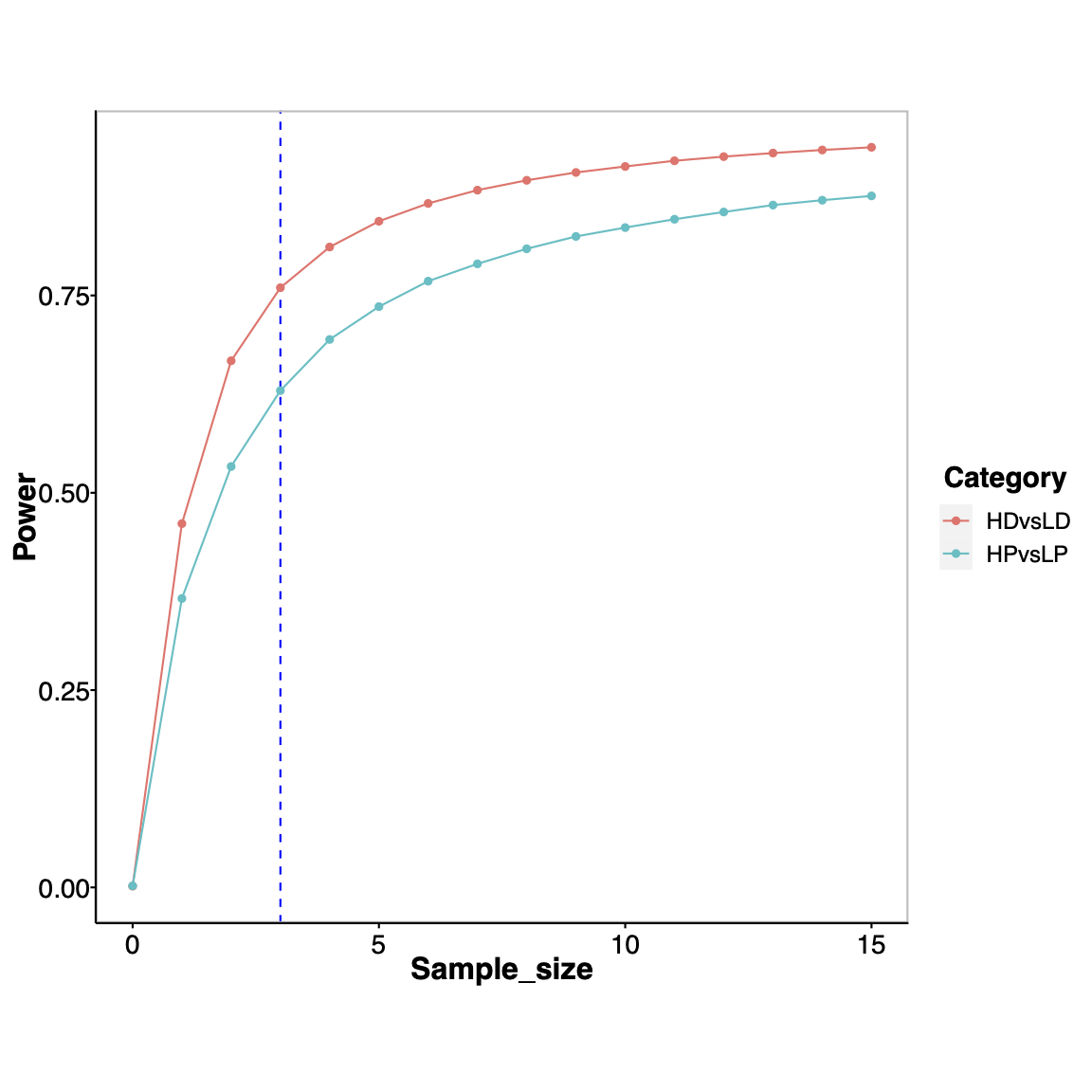

Supplement: Supplementary file 7 [file Image1.TIF]
